# High mortality associated with inappropriate initial antibiotic therapy in hematological malignancies with *Klebsiella pneumoniae* bloodstream infections

**DOI:** 10.1038/s41598-024-63864-5

**Published:** 2024-06-06

**Authors:** Zijun Ma, Chengcheng Lai, Jun Zhang, Yuren Han, Mengjie Xin, Jinghui Wang, Zhuanghao Wu, Yonggang Luo

**Affiliations:** 1 Department of General Practice, The First Affiliated Hospital of Zhengzhou University, Zhengzhou, China; 2 Department of Pharmacy, The First Affiliated Hospital of Zhengzhou University, Zhengzhou, China; 3 Department of Gastroenterology, The First Affiliated Hospital of Zhengzhou University, Zhengzhou, China; 4 Department of Medical Equipment, The First Affiliated Hospital of Zhengzhou University, Zhengzhou, China; 5 Department of General Practice, Luoyang Central Hospital Affiliated to Zhengzhou University, Luoyang, China; 6 Department of Integrated Intensive Care Unit, The First Affiliated Hospital of Zhengzhou University, Zhengzhou, China; 7 Department of Neurosurgical Intensive Care Unit, The First Affiliated Hospital of Zhengzhou University, Zhengzhou, China

**Keywords:** Hematological malignancies, *Klebsiella pneumoniae*, Bloodstream infection, Antibiotic therapy, Carbapenem-resistant, Antimicrobials, Bacteria, Clinical microbiology, Haematological cancer

## Abstract

Bloodstream infections caused by multidrug-resistant organisms such as *Klebsiella pneumoniae* are a significant challenge in managing hematological malignancies. This study aims to characterize the epidemiology of *Klebsiella pneumoniae* bloodstream infections specifically in patients with hematological malignancies, delineate the patterns of initial antibiotic therapy, assess the prevalence of resistant strains, identify risk factors for these resistant strains, and evaluate factors influencing patient outcomes. A retrospective analysis was conducted at a single center from January 2017 to December 2020, focusing on 182 patients with hematological malignancies who developed *Klebsiella pneumoniae* bloodstream infections. We compared the 30-day mortality rates between patients receiving appropriate and inappropriate antibiotic treatments, including the effectiveness of both single-drug and combination therapies. Kaplan–Meier survival analysis and multivariate logistic and Cox regression were used to identify factors influencing mortality risk. The 30-day all-cause mortality rate was 30.2% for all patients. The 30-day all-cause mortality rates were 77.2% and 8.8% in patients who received inappropriate initial treatment and appropriate initial treatment (*p* < 0.001). Inappropriate initial treatment significantly influenced mortality and was a key predictor of 30-day mortality, along with septic shock and previous intensive care unit (ICU) stays. Patients with carbapenem-resistant *Klebsiella pneumoniae* (CRKP) bloodstream infections exhibited more severe clinical symptoms compared to the CSKP group. The study demonstrates a significant association between empirical carbapenem administration and the escalating prevalence of CRKP and multidrug-resistant *K. pneumoniae* (MDR-KP) infections. Furthermore, the study identified inappropriate initial antibiotic therapy, septic shock, and ICU admission as independent risk factors for 30-day mortality.

## Introduction

*Klebsiella pneumoniae* (KP) is a common pathogen causing hospital-acquired bloodstream infections. In recent years, there has been a worrisome emergence of antimicrobial-resistance in Gram-negatives, including KP. Particularly concerning is the rise of carbapenem-resistant *Klebsiella pneumoniae* (CRKP) which has become a significant problem in several countries. Bloodstream infections (BSIs) caused by KP including hypervirulent strains, are a significant complication in patients diagnosed with hematological malignancies (HM)^1^. In these cases, timely and appropriate antimicrobial treatment is crucial. Studies have shown that delays in starting such treatment, or the use of inappropriate antimicrobial regimens, are linked to increased mortality rates^2–7^. Despite the well-recognized importance of early and appropriate antibiotic therapy in managing sepsis, adherence to this principle has been disappointingly low. The present investigation aims to analyze the clinical characteristics and outcomes in HM patients with KP BSI who received initial antimicrobial therapy that was discordant, as indicated by in vitro susceptibility testing. *Klebsiella pneumoniae* is a type of bacteria that can cause serious infections, including bloodstream infections. The treatment of these infections can be challenging, as the bacteria have developed resistance to many antibiotics, particularly in healthcare settings. Individuals with hematological malignancies (HMs) are at increased risk of infectious complications, attributable to an immunocompromised state engendered by either the intrinsic pathology or the cytotoxic repercussions of chemotherapeutic interventions, or a combination thereof^8^. Recent studies have focused on identifying factors that influence susceptibility to such infections, improving diagnostic and therapeutic methods, and developing preventative measures against the spread of antibiotic-resistant strains^9,10^. This area of research, however, has often stopped short of yielding concrete conclusions that can directly guide clinical practice. Given the rising threat of drug-resistant bacteria in patients with hematological malignancies, this study delves deeper into the risk factors, microbial characteristics, and mortality associated with bloodstream infections caused by these resistant pathogens. By analyzing clinical data from a single center, we explored the link between antibiotic use and bacterial resistance. This analysis also identified key factors that influence patient outcomes. Ultimately, this study aims to provide real-world evidence to guide anti-infective treatment for this immunologically compromised population, empowering healthcare professionals to make more informed clinical decisions.

## Methods

### Setting, study population, and design

This retrospective study, conducted at the First Affiliated Hospital of Zhengzhou University, was approved by the Institutional Ethics Committee and was exempt from full review as it used de-identified patient data. A waiver for informed consent was granted by the IRB due to the study's retrospective design and the anonymization of data. All procedures adhered to the ethical standards of the institutional and/or national research committee, the Helsinki declaration, and its subsequent amendments. No animal studies were involved, and patient confidentiality was strictly maintained.

Data were retrospectively gleaned from the electronic medical records of patients admitted to a tertiary care hospital affiliated with a university, located in Henan Province, China, during the period spanning January 2017 to December 2020. All episodes of bloodstream infections caused by *Klebsiella pneumoniae* (KP BSIs) were identified in adult patients with a confirmed diagnosis of hematological malignancies (HM). Included patients received a minimum of one systemic antibiotic either on the day of blood culture collection or the subsequent day, and had available antibiotic susceptibility data. In total, 811 blood culture specimens were obtained from 182 patients diagnosed with HM over the course of the study; of these, 614 samples (75.7%) tested positive for *Klebsiella pneumoniae*. The analysis was restricted to the first episode of bloodstream infection for each patient as recorded in our registry. Prophylactic antibacterial treatment was dispensed to all patients included in this study in alignment with the 2020 edition of the Guidelines for Clinical Use of Antibiotics in Chinese Neutropenia Patients with Fever^11^. Data regarding patients manifesting positive blood cultures were collated, and the subsequent employment of antimicrobial therapies was scrutinized. Exclusion criteria encompassed: (1) absence of a hematologic malignancy diagnosis; (2) contamination of blood culture samples; (3) loss to follow-up; (4) age below 18 years; (5) polymicrobial bacteremia. The principal outcome assessed was the mortality rate within 30 days following the infection. If a detailed flowchart for inclusion and exclusion is required, please refer to Appendix 1.

### Definitions

The following terms were defined before data analysis: KP bloodstream infections were characterized by a positive blood culture for KP accompanied by clinical indicators of systemic inflammatory response syndrome^12^. Inappropriate initial antibiotic therapy (IIAT) was defined as the administration of systemic antibiotics to which the isolated bloodstream pathogen was demonstrated to be resistant, according to its in vitro susceptibility profile as indicated by the blood culture results^13–15^. The assessment of IIAT was circumscribed to individuals who commenced antibiotic therapy on the day of blood culture acquisition, thereby zeroing in on patients with a presumptive diagnosis of either bloodstream infection or sepsis. Additionally, the broader spectrum of activity of the initial antibiotic regimen against *Klebsiella pneumoniae* was evaluated. In instances where the administered antibiotic(s) were not explicitly identified, resistance or susceptibility profiles were extrapolated from analogous agents within the same pharmacologic class^13^. For instance, cefepime, which is a fourth-generation cephalosporin, was considered effective in vitro against an isolate if that isolate had been identified as susceptible to ceftriaxone, a third-generation cephalosporin. Sepsis was delineated per the Sepsis-3 guidelines as BSI concomitant with organ dysfunction, quantified by an elevation in the Sequential Organ Failure Assessment (SOFA) score by at least two points from baseline, as previously described^16^. Septic shock was characterized as the occurrence of BSI concomitant with the administration of at least one vasopressor agent within 24 h of blood culture collection^17^. Neutropenia was defined as an absolute neutrophil count (ANC) less than 500 neutrophils/μL at the initiation of BSI; the neutropenic state was deemed to be prolonged if it persisted for 10 days or more, and was classified as severe if the ANC dropped below 100 neutrophils/μL^14^.

### Microbiological methods

Antibiotic susceptibility assays were executed on the isolated bacterial strains employing agar dilution and broth microdilution techniques. The panel of antimicrobial agents assessed comprised amikacin, aztreonam, ceftriaxone, cefoxitin, levofloxacin, sulfamethoxazole, cefepime, piperacillin-tazobactam, cefperazone-sulbactam tigecycline, and polymyxin B. Interpretations of tigecycline and polymyxin B susceptibility were carried out in alignment with the criteria and recommendations delineated by the European Committee on Antimicrobial Susceptibility Testing (EUCAST, 2019). Susceptibility profiles for the remaining antimicrobial agents were ascertained in conformity with the guidelines established by the Clinical and Laboratory Standards Institute (CLSI, 2019). E. coli ATCC 25,922 served as the quality control strain for the antibiotic susceptibility assays.

### Statistical analysis

Continuous variables were analyzed using the Student's t-test for normally distributed data and the Mann–Whitney *U* test for non-normally distributed data. Categorical variables were analyzed using the Chi-square test or Fisher's exact test, depending on the data distribution. Univariate logistic regression was performed initially to identify potential predictors. Odds ratios (ORs) with 95% confidence intervals (CIs) were calculated to determine the statistical significance of observed associations. Variables with a *p* value < 0.2 in univariate analysis or those deemed clinically significant were considered for inclusion in the multivariate analysis. Continuous variables are expressed as means ± SD, while categorical variables are shown as percentages within their respective groups. A *p* value < 0.05 from two-tailed tests was considered statistically significant. Multivariate logistic regression was conducted to identify independent risk factors for mortality at day 30. When using logistic or Cox regression, either ORs or HRs were calculated, accompanied by their 95% *CI*s. The survival curves were plotted with the Kaplan–Meier method. All statistical analysis was conducted using R 4.3.1 or SPSS version 26.0.

### Ethics approval and consent to participate

This study, conducted at the First Affiliated Hospital of Zhengzhou University, was approved by the Institutional Ethics Committee (Approval Number: 2022-KY-1269). It was exempt from full review and informed consent due to the use of de-identified data and its retrospective nature, as IRB guidelines. The study adhered to the ethical standards of the Declaration of Helsinki and ensured strict confidentiality of patient information. No animal studies were involved.

## Results

A total of 182 episodes of bloodstream infections due to *Klebsiella pneumoniae* were investigated in this study. Among these cases, resistance to carbapenems was detected in 44.0% (n = 80) of the KP isolates. The clinical and epidemiological attributes of patients, segregated based on the carbapenem susceptibility profile of their *Klebsiella pneumoniae* isolates, are comprehensively delineated in Table 1.

**Table 1:** Comparative clinical and demographic characteristics of BSI Caused by CRKP and CSKP in patients with HM.

Variables	CSKP, N = 102^a^	CRKP, N = 80^a^	*p* value^b^
Demographic information			
Male sex	48 (47%)	47 (59%)	0.2
Age	50 (34, 58)	46 (34, 56)	0.8
Combined infection			
Pneumonia	50 (49%)	48 (60%)	0.2
Skin and skin structures	3 (2.9%)	7 (8.8%)	0.2
Cholecystitis	1 (1.0%)	3 (3.8%)	0.4
Perianal infection	5 (4.9%)	18 (22%)	< 0.001
Clinical Scoring Systems			
Charlson comorbidity index score	27 (26%)	32 (40%)	0.076
SOFA	4.0 (4.0, 6.0)	10.5 (5.0, 14.0)	< 0.001
Comorbidities			
Myocardial infarction	2 (2.0%)	2 (2.5%)	> 0.9
Congestive heart failure	4 (3.9%)	15 (19%)	0.003
Cerebrovascular disease	3 (2.9%)	4 (5.0%)	0.7
Chronic pulmonary disease	1 (1.0%)	1 (1.3%)	> 0.9
Peptic ulcer disease	2 (2.0%)	1 (1.3%)	> 0.9
Severe hepatic disease	3 (2.9%)	2 (2.5%)	> 0.9
Diabetes	5 (4.9%)	4 (5.0%)	> 0.9
Renal disease	5 (4.9%)	7 (8.8%)	0.5
Solid tumour	2 (2.0%)	2 (2.5%)	> 0.9
Drinking^c^	6 (5.9%)	3 (3.8%)	0.8
Smoking^c^	11 (11%)	7 (8.8%)	0.8
Healthcare exposures within 30 days of BSI			
Chemotherapy	77 (75%)	62 (78%)	0.9
Surgery	0 (0%)	5 (6.2%)	0.035
Indwelling CVC	13 (13%)	15 (19%)	0.4
Characteristics at time of BSI			
Duration of admission	48 (33, 57)	46 (34, 56)	0.8
ICU	18 (18%)	50 (62%)	< 0.001
Duration of neutropenia	10 (5, 18)	14 (8, 19)	0.2
ANC < 100 neutrophils/μL	99 (97%)	80 (100%)	0.3
ANC < 500 neutrophils/μL for at least 10 days	76 (75%)	62 (78%)	0.8
PCT	7.1 (0.4,25.1)	2.4 (0.4,16.5)	0.192
CRP	129.8 (78.4, 210.3)	112.0 (77.6, 176.6)	0.493
LDH	152 (118.0, 270.0)	223.5 (144.8, 418.8)	0.071
Mechanical ventilation	10 (9.8%)	20 (25%)	0.011
Receiving glucocorticoids	76 (75%)	67 (84%)	0.2
Diarrhoea	10 (9.8%)	12 (15%)	0.4
Anal fissure	4 (3.9%)	4 (5.0%)	> 0.9
Haematological malignancy			0.065
Acute myeloid leukaemia	61 (60%)	57 (71%)	
Acute lymphatic leukaemia	29 (28%)	14 (18%)	
Lymphoma	0 (0%)	3 (3.8%)	
Multiple Myeloma	2 (2.0%)	0 (0%)	
Myelodysplastic syndrome	10 (9.8%)	6 (7.5%)	
State of Haematological Disease			0.521
Newly diagnosed	20 (20%)	22 (28%)	
Complete remission	14 (14%)	11 (14%)	
Refractory	6 (5.9%)	1 (1.3%)	
Relapsed	6 (5.9%)	8 (10%)	
HSCT	6 (5.9%)	4 (5.0%)	
Unknown	3 (2.9%)	2 (2.5%)	
Uncontrolled	47 (46%)	32 (40%)	
Presentation of BSI			
Pitt bacteraemia score	2.0 (1.0, 2.0)	6.0 (2.0, 10.0)	< 0.001
Septic shock	24 (24%)	54 (68%)	< 0.001
Hypotension	23 (23%)	58 (72%)	< 0.001
Outcomes			
7-day mortality	14 (14%)	5 (6.2%)	0.2
30-day mortality	12 (12%)	43 (54%)	< 0.001
Mortality during index hospitalization	15 (15%)	49 (61%)	< 0.001
BSI-related mortality	9 (8.8%)	46 (57%)	< 0.001
Antibiotic resistance by KP isolate			
Third generation cephalosporins	23 (23%)	80 (100%)	< 0.001
Fourth generation cephalosporins	6 (5.9%)	78 (98%)	< 0.001
Fluoroquinolones	20 (20%)	73 (91%)	< 0.001
Piperacillin/tazobactam	4 (3.9%)	78 (98%)	< 0.001
Tigecycline	4 (3.9%)	7 (8.8%)	0.3
Polymyxins	0 (0%)	3 (3.8%)	0.2
Antibiotic resistance			
Non-MDR	88 (86%)	0 (0%)	
MDR	14 (14%)	11 (14%)	
XDR	0 (0%)	66 (82%)	
PDR	0 (0%)	3 (3.8%)	
Empirical anti-infection treatment			
Carbapenems	27 (26%)	60 (75%)	< 0.001
Cephalosporins	8 (7.8%)	22 (28%)	< 0.001
Tigecycline	6 (5.9%)	16 (20%)	0.008
Polymyxin	0 (0%)	4 (5.0%)	0.076
Fluoroquinolones	13 (13%)	31 (39%)	< 0.001
Piperacillin/tazobactam	18 (18%)	24 (30%)	0.074
Initial anti-infection treatment			
Inappropriate initial therapy	1 (1.0%)	56 (70%)	< 0.001
Combination initial antibiotic therapy	36 (35%)	52 (65%)	< 0.001

^a^Median (IQR); n (%).

^b^Welch Two Sample t-test; Two sample test for equality of proportions; Standardized Mean Difference.

^c^“Smoking” and “drinking” describe the historical consumption of cigarettes and alcoholic beverages by patients. The average annual cumulative consumption is 16,816 cigarettes × year for smoking and 76,509 ml × year for drinking, with both the median and quartiles at 0. Mann–Whitney U test analysis showed no significant differences in the distribution of cumulative smoking (*p* = 0.646) and drinking amounts (*p* = 0.534) between the groups.

When compared to patients afflicted with carbapenem-susceptible KP (CSKP) BSI, individuals with carbapenem-resistant KP (CRKP) BSI exhibited a higher PBS and SOFA score, and were more likely to have a prior history of intensive care unit (ICU) admission, prior mechanical ventilation, and receipt of antibiotic prophylaxis, particularly with carbapenems or cephalosporins.

**Figure 1:**
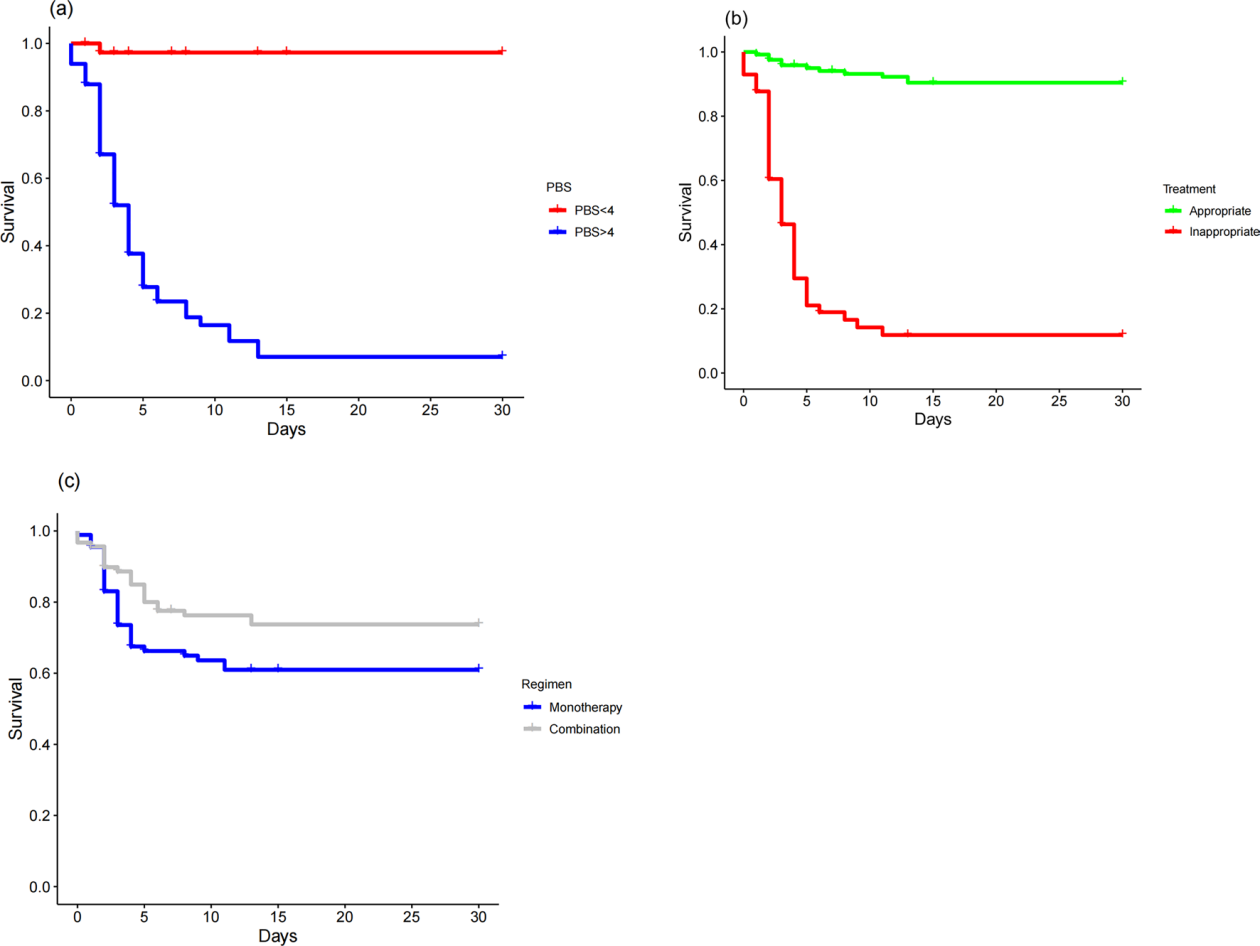
Kaplan–Meier univariate survival estimates. (**a**) PBS within 48 h before or on the day of first positive blood culture; *p* < 0.05. (**b**) (In)appropriate initial antibiotic therapy after the bloodstream infection; *p* < 0.05. (**c**) Anti-KP therapeutic regimen; *p* = 0.058.

The cumulative 30-day mortality rate stood at 30% (n = 55/182). Nevertheless, a significantly elevated mortality rate was noted in patients with CRKP BSI (54%, n = 43/80) as opposed to those with BSI due to CSKP (12%, n = 12/102; *p* < 0.001). Survival curve analysis underscored the increased mortality risk associated with PBS ≥ 4, inappropriate initial treatment (*p* < 0.0001) (Fig. 1).

### Demographics and epidemiology

The participants ranged in age from 18 to 79 years, with a median age of 48. There was no statistically significant difference in age between the CSKP and CRKP groups, with respective median ages of 50 and 46 years (*p* = 0.8). Similarly, the gender distribution was balanced overall, with 48% females (n = 87) and 52% males (n = 95). In subgroup analysis, the CSKP group was 53% female (n = 54) and 47% male (n = 48), while the CRKP group was 41% female (n = 33) and 59% male (n = 47). The difference in gender distribution between the two groups was not statistically significant (*p* = 0.24).

In terms of clinical manifestations and comorbidities, fever was a common initial symptom. Septic shock subsequent to BSI was noted in 43% of the participants. A range of combined infections were observed, including pneumonia in 54% (n = 98), skin and skin structure infections in 5.5% (n = 10), cholecystitis in 2.2% (n = 4), and perianal infections in 13% (n = 23). Additional manifestations included oral mucositis in 10 individuals and pulmonary symptoms in 108 participants, aligning with the high rate of pneumonia.

The most prevalent underlying hematologic malignancy was acute myeloid leukemia (AML), making up 64.8% (n = 118) of the participants. This was followed by acute lymphoblastic leukemia (ALL) at 23.6% (n = 43), myelodysplastic syndromes (MDS) at 8.8% (n = 16), lymphoma at 1.6% (n = 3), and multiple myeloma at 1.1% (n = 2). Of the total participants, 139 had undergone chemotherapy and 5 were recipients of hematopoietic stem cell transplantation (HSCT).

### Antimicrobial susceptibility of the isolates

**Figure 2:**
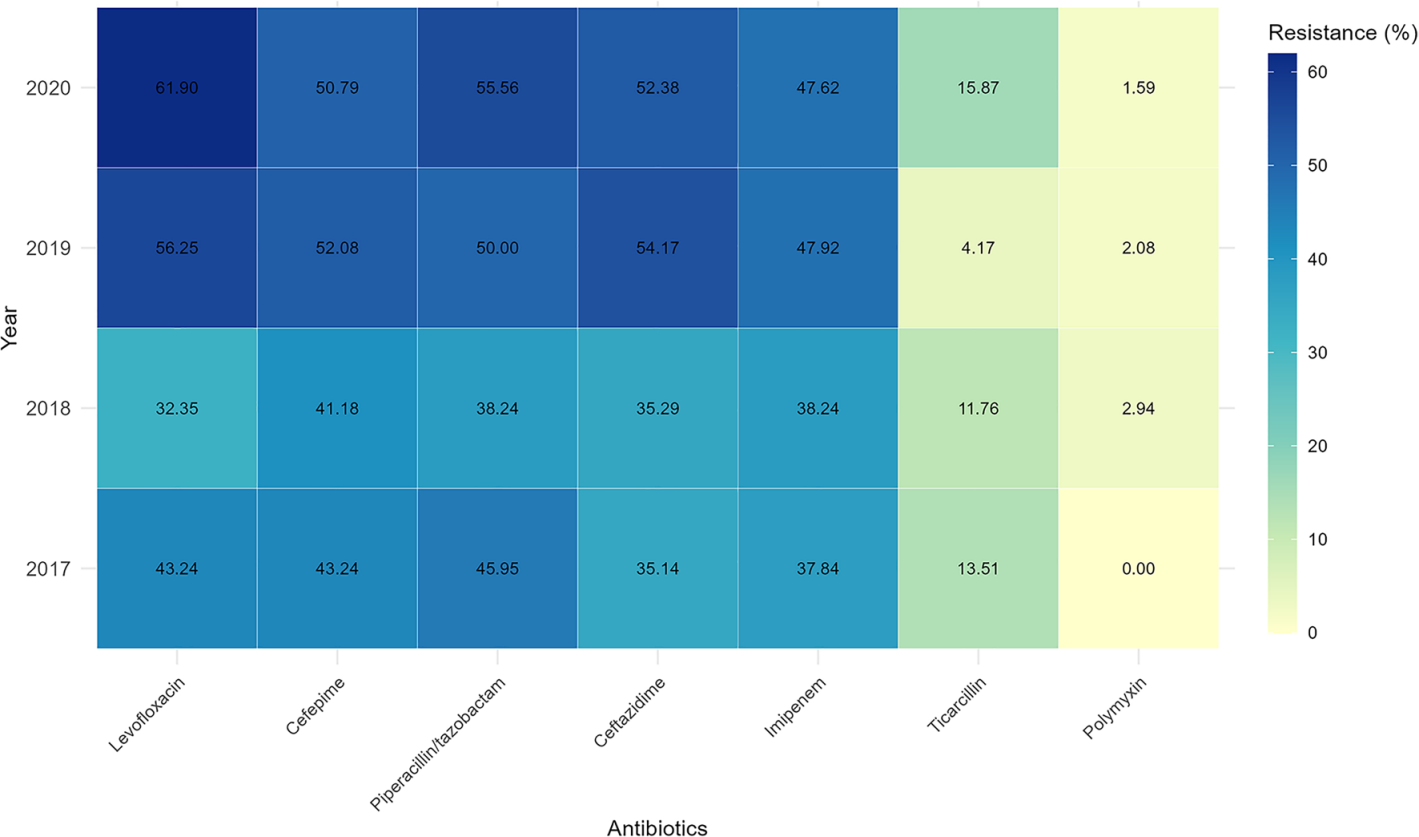
Yearly changes in antibiotic resistance rate.

Of the 182 strains isolated, Polymyxin showcased the highest antimicrobial activity against these strains, boasting an efficacy of 96.8%, as detailed in Fig. 2. This was closely followed by ticarcillin and imipenem, with efficacies of 88.5% and 56.0%, respectively. Notably, even among the carbapenem-resistant strains, some remained sensitive to specific alternative β-lactams, such as ceftazidime, and a variety of non-β-lactam antimicrobials.

We embarked on a comprehensive analysis of the annual antibiotic resistance trends in CRKP samples over a four-year period, from 2017 to 2020. This involved calculating the annual resistance proportions for each antibiotic and conducting a trend analysis to understand the temporal changes in these proportions. Our findings revealed positive slopes for all antibiotics, indicating an increase in resistance proportions over the years. However, the significance of these trends varied: while most showed *p* values exceeding the threshold of 0.05, indicating a lack of statistical significance, there were notable exceptions. Specifically, the slopes for Cefepime, Ceftazidime, Gentamicin, Imipenem, Levofloxacin, Piperacillin/tazobactam, Polymyxin, and Ticarcillin were 0.032 (*p* = 0.330), 0.067 (*p* = 0.039), 0.021 (*p* = 0.510), 0.038 (*p* = 0.250), 0.081 (*p* = 0.013), 0.043 (*p* = 0.190), 0.0032 (*p* = 0.710), and 0.0051 (*p* = 0.810) respectively. The statistically significant slopes for Ceftazidime and Levofloxacin are particularly noteworthy, suggesting an upward trend in resistance, while the others, despite showing an upward trend, do not have strong statistical backing. In our detailed exploration into resistance patterns, for the MDR group, the antimicrobial susceptibility rates for aminoglycosides, cephalosporins, piperacillin/tazobactam, fluoroquinolones, and carbapenems stood at 13.8%, 12.7%, 8.5%, 8.5%, and 14.8%, respectively. Conversely, within the CR group, the rates were noted as 10.0%, 7.5%, 0.0%, 8.7%, and 0.0%, respectively. After adjusting for potential confounders using multivariate logistic regression analysis, certain determinants emerged as potentially influencing MDR. Perianal infection was strongly associated with increased odds of MDR (OR = 13.25, 95% CI: 2.72, 64.69, *p* < 0.001). Empirical administration of carbapenems and empirical use of fluoroquinolones both had statistically significant relations with MDR, with ORs of 4.13 and 3.18, respectively. Other factors such as age, sex, Pitt bacteremia score, days of neutropenia surpassing 15, pneumonia, prior mechanical ventilation use, CCI, SOFA, empirical administration of cephalosporin, empirical use of tigecycline, empirical administration of polymyxin, and empirical usage of enzyme inhibitors did not show a marked relationship with MDR. Given the complexity of this domain, it's essential to consider that even after accounting for these potential confounders in our multivariate analysis, there remains a potential risk of residual confounding from unobserved variables.

### Risk factors for acquisition of CRKP BSI and MDR-KP

Upon univariate analysis (Table 2), several risk factors were identified to contribute significantly to the incidence of KP BSI caused by CRKP. These included prolonged neutropenia (exceeding 10 days), perianal infection, increased Charlson comorbidity index score, elevated SOFA score, bacteremia score, hypotension, hypoalbuminemia, and previous usage of antibiotics including carbapenems, cephalosporins, tigecycline, and fluoroquinolones. The existence of a perianal infection, the presence of congestive heart failure, hypotension, and previous utilization of carbapenems were markedly associated with a heightened risk of CRKP-induced bloodstream infection. These findings imply that these elements might independently affect the probability of encountering BSI caused by CRKP, maintaining their significance even after accounting for other variables in the analysis.

**Table 2:** Logistic regression analysis of risk factors for CRKP BSI development.

Variable	Univariate		Multivariate^c^	
	OR (95% CI)^a^	*p* value^b^	OR (95% CI)^a^	*p* value^b^
Neutropenia days ≥ 10	1.72 (0.94–3.13)	0.0782	1.35 (0.56–3.25)	0.509
Perianal infection	5.63 (1.99–15.95)	< 0.001	10.87 (2.64–44.84)	0.00096
Charlson comorbidity index score	1.41 (1.03–1.92)	0.03	0.89 (0.57–1.4)	0.612
Congestive heart failure	5.65 (1.8–17.8)	< 0.001	4.84 (1.07–21.85)	0.0404
Previous admission in ICU	7.78 (3.94–15.37)	< 0.001	2.03 (0.64–6.41)	0.229
Previous used mechanical ventilation	3.07 (1.34–7)	0.01	0.63 (0.13–2.99)	0.562
Pitt bacteremia score	1.24 (1.14–1.35)	< 0.001	1.02 (0.85–1.22)	0.817
Hypotension	9.06 (4.61–17.8)	< 0.001	4.04 (1.05–15.48)	0.0417
Hypoalbuminemia	5.18 (2.71–9.91)	< 0.001	2.49 (0.99–6.29)	0.0536
Empirical anti-infection treatment				
Carbapenems	8.33 (4.26–16.29)	< 0.001	5.81 (2.29–14.74)	0.00021
Cephalosporins	4.46 (1.86–10.67)	< 0.001	1.74 (0.54–5.6)	0.35
Tigecycline	4 (1.49–10.77)	0.01	0.73 (0.17–3.09)	0.671
Fluoroquinolones	4.33 (2.08–9.04)	< 0.001	1.93 (0.73–5.06)	0.183

^a^OR: Odds Ratio; CI: Confidence Interval.

^b^*p* < 0.05 for significance.

^c^The Variance Inflation Factor (VIF) values are all relatively low, with none exceeding the common threshold of 10. The pseudo R-squared value is approximately 0.414.

Conducted multivariate analysis elucidated significant risk factors impacting the onset of MDR KP BSI in HM patients (Appendix 2).Perianal infection (OR 11.78 [95% CI 2.80–49.56], *p* < 0.001), congestive heart failure (OR 7.28 [95% CI 1.64–32.31], *p* = 0.009), prior administration of carbapenems (OR 5.61 [95% CI 2.46–12.81], *p* < 0.001), and earlier usage of fluoroquinolones (OR 4.15 [95% CI 1.49–11.55], *p* = 0.007) were all significantly linked with an augmented risk of MDR-related KP BSI. Such insights underscore the significance of these factors in the pathogenesis of MDR-induced KP BSI.

### Treatments

Following blood culture sampling, all patients were promptly administered initial antimicrobial therapy, aligning with standard clinical protocols. Among the 182 patients, 125 (68.7%) were administered appropriate initial therapy within 48 h of BSI onset, reflecting adherence to recommended treatment timelines. Of the 125 patients who received appropriate initial therapy, 68 (54.4%) were treated with monotherapy, while 57 received combination therapy. Conversely, 57 patients received initial treatment that was later determined to be inappropriate. Out of the 57 patients subjected to inappropriate treatment, 56 were infected with CR strains.

In the initial monotherapy group, the majority of patients (78 patients, or 84.8%) were treated with carbapenems, followed by compound preparations of β-lactamase inhibitors (5.4%), tigecycline (4.3%), cephalosporins (3.3%), and fluoroquinolones (2.2%). Among the 90 patients who received combination therapy initially, most were treated with two-drug regimens. More specifically, combinations of carbapenems with tigecycline accounted for 44.4% of these treatments, followed by carbapenems plus fluoroquinolones (12.2%), and carbapenems with polymyxin (7.8%). Three-drug combinations were utilized less frequently, with the polymyxin, carbapenems, and tigecycline regimen accounting for 10% of such treatments. Complete data on specific drug counts, percentages, and combinations are detailed in Appendix 3.

Antibiotic regimens following initial treatment were categorized into four scenarios based on the results of positive blood cultures: Maintain initial regimen (32.4%): This group of patients continued with the initial antibiotic regimen they were receiving when their blood cultures became positive. Escalate therapy (20.9%): In this group, the antibiotic regimen was intensified either by increasing the number of antibiotics or by broadening the spectrum of antibiotics from narrow-spectrum to broad-spectrum. De-escalate therapy (13.2%): For this group, the antibiotic regimen was simplified either by reducing the number of antibiotics or by narrowing the spectrum of antibiotics from broad-spectrum to narrow-spectrum. Data on subsequent antibiotic regimens unavailable due to discharge or death (33.5%): For this group of patients, information on subsequent antibiotic regimens was not available due to either discharge from the hospital or death.

### Risk factors for 30-day mortality in patients with KP BSI

In a univariate Cox regression analysis examining factors associated with 30-day mortality in patients with KP BSI, several variables demonstrated a statistically significant association. These include initial antimicrobial therapy deemed inappropriate (Hazard Ratio [HR] 16; 95% Confidence Interval [CI], 8.3–32; *p* < 0.001), previous admission to an Intensive Care Unit (ICU) (HR 16; 95% CI 8–31; *p* < 0.001), antecedent surgical procedures (HR 5.1; 95% CI 2–13; *p* < 0.001), bloodstream infections attributable to CRKP (HR 6.7; 95% CI 3.5–13; *p* < 0.001), hypoalbuminemia (HR 3.7; 95% CI 2.1–6.3; *p* < 0.001), elevated Pitt bacteremia scores (HR 1.3; 95% CI 1.3–1.4; *p* < 0.001), septic shock (HR 43; 95% CI 13–140; *p* < 0.001), and Sequential Organ Failure Assessment (SOFA) scores (HR 1.4; 95% CI 1.3–1.5; *p* < 0.001) as delineated in Appendix 4.

Concomitantly, the multivariate cox regression analysis delineated pivotal prognostic factors for 30-day mortality as presented in Table 3. These encompassed septic shock (HR 12.56; 95% CI 3.45–45.71; *p* < 0.001), inappropriate initial antimicrobial therapy (HR 4.48; 95% CI 2.17–9.23; *p* < 0.001), and prior ICU stay (HR 2.49; 95% CI 1.19–5.22; *p* = 0.016).

**Table 3:** Multivariate cox regression analysis of variables associated with 30-day Mortality.

Variables	HR (95% CI)	*p* value
Inappropriate initial antimicrobial therapy	4.48 (2.17–9.23)	< 0.001
Septic shock	12.56 (3.45–45.71)	< 0.001
ICU	2.49 (1.19–5.22)	0.016

Concordance = 0.883; Likelihood Ratio, Wald, and Score Tests: All these tests have highly significant *p* values (< 0.0001), indicating that the model as a whole fit the data significantly better than a model with no predictors.

## Discussion

According to the China Bacterial Resistance Monitoring Report from October 2021 to September 2022, the isolation rate of *Klebsiella pneumoniae* was 21.2%, and the national average carbapenem resistance rate was 10.0%, with Henan Province reaching 19%^18^. In this study, the carbapenem resistance rate of *Klebsiella pneumoniae* strains isolated from acute leukemia patients was 44%, which is consistent with previous studies^15,19,20^. Patients with hematologic malignancies have a higher risk of infection with resistant bacteria. The possible reasons are the increase of malignant cells due to the disease itself, the suppression of the immune system and gastrointestinal toxicity during the treatment process (such as chemotherapy and bone marrow transplantation), which increase the risk of hospital-acquired multidrug-resistant (MDR) bacterial infection in these patients^21–23^. The difficulty in treating MDR infections may lead to delay or interruption of disease treatment, affect the treatment effect of the malignancy, increase the recovery time of patients, and therefore, the mortality rate of hematologic malignancies combined with bloodstream infection is higher than that of other populations^24^. Our study emphasizes the critical role of appropriate targeted therapeutic decision-making in effectively managing the complex challenges presented by HM and BSI caused by CRKP. Patients with malignant hematologic disorders are at a higher risk of bloodstream infections due to a compromised immune system, side effects of radiotherapy and chemotherapy, neutropenia, frequent hospitalizations, and invasive medical procedures^25^.

Bloodstream infections cause prolonged hospital stays, increase direct medical costs for patients, and result in a considerably high mortality rate^26^. To prevent CRKP bloodstream infections in patients with hematologic malignancies, it is crucial to implement comprehensive infection control strategies. These include strict adherence to hand hygiene protocols, rigorous environmental cleaning, and disinfection routines^27^. Active surveillance for perianal infections and prompt intervention when identified is also vital^28^. Additionally, judicious use of antimicrobials through an antimicrobial stewardship program, along with raising public awareness about the serious situation of antibiotic resistance, can mitigate the development of resistance^29–32^.

In our study, perianal infection, congestive heart failure, hypotension and a history of carbapenem use were identified as independent risk factors for CRKP bloodstream infection in patients with hematological malignancies. The presence of a perianal infection suggests potential colonization with CRKP, which can act as a reservoir for the bacteria and facilitate its spread into the bloodstream. Retrospective analysis of the patients' medical records revealed a 3.3% (6/182) prevalence of intestinal colonization with KP based on pre-blood culture test data. It is important to note that these prevalence rates may be underestimated, as anal swab testing was not performed on all patients. Both congestive heart failure and hypotension (low blood pressure) can weaken the body's defenses against infection. They achieve this by impairing blood circulation and organ perfusion, which limits the ability of immune cells and antibiotics to reach the infection site. Prior exposure to carbapenems, particularly prolonged or inappropriate use, can contribute to the development of CRKP resistance. The current findings corroborate the outcomes of prior studies^33^. Therefore, ensuring optimal nutritional status and managing comorbid conditions effectively are also important components of reducing the risk of CRKP infections in this vulnerable patient population. Interventions focusing on the rapid detection of carbapenem resistance could be pivotal in reducing the observed 54% mortality rate in CRKP infections. Early identification and management of patients at high risk of BSI caused by CRKP may reduce mortality^34^, which would be a significant improvement in clinical outcomes. Incorporating routine SOFA score assessments in the management of HM patients with KP BSI may facilitate earlier recognition of high-risk patients, enabling more aggressive and targeted interventions. The prognostic value of SOFA scores in bacteremia could be crucial in guiding clinical decisions and improving the stratification of patient care. Our findings indicate a critical need for the implementation of robust antibiotic stewardship programs and the integration of rapid diagnostic assays to reduce the high rate of inappropriate initial therapy in CRKP BSI cases. Such initiatives could minimize the administration of ineffective initial therapy and are essential in the context of rising antimicrobial resistance.

Regarding treatment strategies, current literature suggests a preference for longer courses of synergistic treatment (≥ 10 to 14 days) for more resistant infections, while shorter courses (≤ 5 to 7 days) may be reserved for less resistant Gram-negative bacterial infections^35^. However, our study did not find a significant difference in mortality between monotherapy and combination therapy in HM patients with KP BS. This observation, consistent in our subgroup analysis targeting CRKP, indicates that the choice between monotherapy and combination therapy for initial treatment requires further exploration. Considering patient-specific factors and adhering to antimicrobial stewardship principles is crucial in therapy selection, emphasizing personalized treatment decisions^2–6^.

Our study suggests that patients with CSKP BSI have higher median levels of C-Reactive Protein (CRP, 129.8) and Procalcitonin (PCT, 7.1) compared to patients with BSI caused by CRKP, whose median values for PCT and CRP are 2.4 and 112.0, respectively. Furthermore, Lactate Dehydrogenase (LDH) levels are increased in the CRKP group (median 223.5) versus the CSKP group (median 152). None of these differences reached statistical significance, with *p* values for PCT, CRP, and LDH being 0.192, 0.493, and 0.071, respectively. Given that our study observed no marked differences in clinical symptoms, inflammatory markers, and laboratory indicators between CRKP and CSKP patients, further research and discussion are warranted to determine whether monotherapy or combination therapy should be used in the initial treatment. It is crucial to consider patient-specific factors and antimicrobial stewardship principles when selecting the appropriate therapy for HM patients with BSI, emphasizing the importance of individualized treatment decisions.

The retrospective nature of our study introduces inherent limitations, such as potential confounders and biases. Future research should employ advanced statistical methods like propensity score matching or machine learning to enhance the robustness of findings. These methods could provide a deeper understanding of the factors influencing mortality in HM patients with KP BSI.

Recognizing the limitations inherent to single-center studies, we advocate for future multicenter collaborations coupled with advanced statistical methods. These efforts could provide a more comprehensive understanding of the factors influencing mortality in HM patients with KP BSI, helping to validate and expand upon these findings. Lastly, given the heterogeneity in clinical presentations and outcomes among HM patients with BSI, individualized treatment decisions become paramount. Future investigations should focus on developing algorithms or decision-support tools that integrate patient-specific factors such as disease stage, comorbid conditions, and previous antibiotic exposure. This approach could guide clinicians in selecting the most appropriate therapy, balancing efficacy with the risk of fostering further resistance.

## Conclusion

Patients with CRKP bloodstream infections exhibited more severe clinical symptoms compared to those in the CSKP group. Notably, our study suggests a correlation between empirical carbapenem use and the increased prevalence of both CRKP and MDR-KP infections. Additionally, we identified perianal infection, congestive heart failure, and hypotension as independent risk factors for CRKP. Furthermore, inappropriate initial antibiotic therapy, septic shock, and ICU admissions were determined to be independent risk factors for 30-day mortality.

### Supplementary Information

Supplementary Information.

## Data Availability

Data is provided within the supplementary information files. The datasets from this study are available from the corresponding author on request.

## Abbreviations

ANC: Absolute neutrophil count
BSI: Bloodstream infection
CRKP: Carbapenem-resistant *Klebsiella pneumoniae*
CRP: C-reactive protein
CVC: Central venous catheter
HM: Hematological malignancies
HSCT: Hematopoietic stem cell transplantation
ICU: Intensive care unit
IIAT: Inappropriate initial antibiotic therapy
KP: *Klebsiella pneumoniae*
LDH: Lactate dehydrogenase
MDR: Multidrug-resistant
PBS: Pitt bacteremia score
PCT: Procalcitonin
PDR: Pandrug-resistant bacteria
SOFA: Sequential Organ Failure Assessment
XDR: Extensively drug-resistant bacteria
