# Supplementary material for: High mortality associated with inappropriate initial antibiotic therapy in hematological malignancies with Klebsiella pneumoniae bloodstream infections
Source: Sci Rep. 2024 Jun 6;14:13041. doi: 10.1038/s41598-024-63864-5 (PMC11156844; doi:10.1038/s41598-024-63864-5)
Supplement: Supplementary file 1 — Supplementary Information. [file 41598_2024_63864_MOESM1_ESM.docx]

Appendix


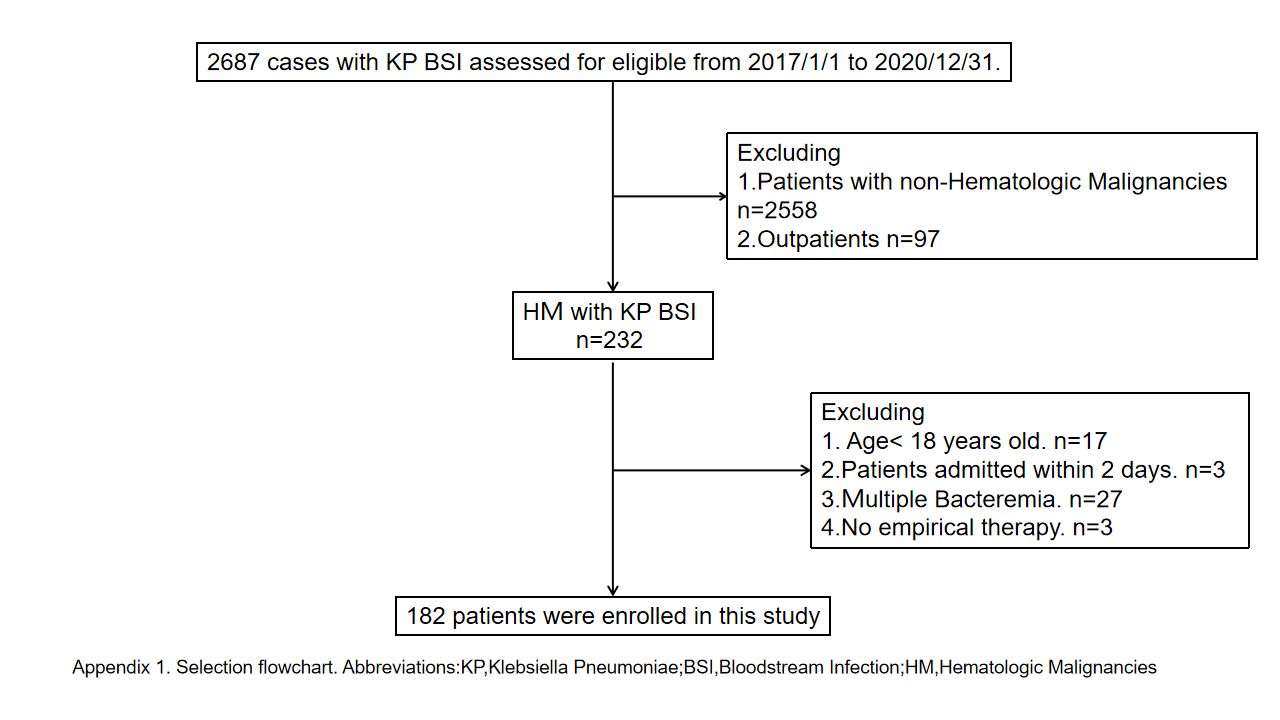


Appendix 1. Selection flowchart.

KP, Klebsiella Pneumoniae; BSI, Bloodstream Infection; HM, Hematologic Malignancies

| **Appendix 2a. Risk Factors for HM patients with BSI Caused by MDR-KP.** | | | | |
| --- | --- | --- | --- | --- |
|  | **Univariate** | | **Multivariate** | |
| **Variable** | **OR (95% CI)** | **p-Value** | **OR (95% CI)** | **p-Value** |
| Neutropenia days ≥10 | 3.33 (1.72-6.71) | *<0.001* | 2.4 (0.87-6.83) | 0.09 |
| Perianal infection | 7.66 (2.5-33.43) | *<0.001* | 18.18 (4.13-104.45) | *<0.001* |
| Charlson comorbidity index score | 1.57 (1.14-2.28) | *0.01* | 1.05 (0.64-1.78) | 0.85 |
| Congestive heart failure | 5.81 (1.85-25.67) | *0.01* | 4.91 (1.02-29.57) | 0.06 |
| Previous admission in ICU | 8.14 (4.07-17.24) | *<0.001* | 2.1 (0.58-7.59) | 0.25 |
| Previous used mechanical ventilation | 3.75 (1.59-9.93) | *<0.001* | 0.79 (0.14-4.37) | 0.79 |
| Pitt bacteraemia score | 1.25 (1.15-1.38) | *<0.001* | 1.03 (0.85-1.25) | 0.79 |
| Hypotension | 7.9 (4.1-15.84) | *<0.001* | 2.94 (0.71-12.41) | 0.14 |
| Hypoalbuminemia | 4.61 (2.43-9.08) | *<0.001* | 1.9 (0.71-5.06) | 0.2 |
| Empirical anti-infection treatment |  |  |  |  |
| Carbapenems | 8.44 (4.39-16.82) | *<0.001* | 4.39 (1.78-11.31) | *<0.001* |
| Cephalosporins | 6.01 (2.36-18.56) | *<0.001* | 2.06 (0.55-8.35) | 0.29 |
| Tigecycline | 11.62 (3.25-74.33) | *<0.001* | 3.03 (0.46-29.8) | 0.29 |
| Fluoroquinolones | 6.21 (2.81-15.27) | *<0.001* | 2.6 (0.92-7.81) | 0.08 |
| Piperacillin tazobactam | 3.44 (1.65-7.68) | *<0.001* | 1.69 (0.52-5.48) | 0.38 |

| **Appendix 2b. Risk Factors for MDR-KP BSI Based on Multivariate Analysis.** | | |
| --- | --- | --- |
| **Variable** | **OR (95% CI)** | **P-Value** |
| Perianal infection | 10.71(2.72-42.17) | <0.001 |
| Congestive heart failure | 5.86(1.38-24.88) | 0.02 |
| Previous use of carbapenems | 6.67(3.17-14.05) | <0.001 |
| Previous use of fluoroquinolones | 3.64(1.4-9.47) | 0.01 |
| The logistic regression model shows moderate explanatory power, with a pseudo R-squared of 0.29 and Nagelkerke's R-squared of 0.44, indicating a significant but not comprehensive explanation of the outcome by the predictors. | | |

| **Appendix 3. Initial treatment therapy for HM patient with KP BSI.** | | | |
| --- | --- | --- | --- |
| **Monotherapy** | **Quantity** | **Combination therapy** | **Quantity** |
| Carbapenems | 78(42.9%) | Carbapenems+Tigecycline | 40(22%) |
| β-lactamase inhibitors | 5(2.7%) | Carbapenems+Fluoroquinolones | 11(6%) |
| Cephalosporins | 3(1.6%) | Carbapenems+Polymyxin | 7(3.8%) |
| Tigecycline | 4(2.2%) | Polymyxin+Carbapenems+Tigecycline | 9(4.9%) |
| Fluoroquinolone | 2(1.1%) | Cephalosporin+Carbapenems+Tigecycline | 2(1.1%) |
|  |  | Carbapenems+Aminoglycoside | 2(1.1%) |
|  |  | Carbapenems+β-lactamase inhibitors | 1(0.5%) |
|  |  | Carbapenems+Azactam | 1(0.5%) |
|  |  | Carbapenem+Tigacycline+Fosfomycin | 1(0.5%) |
|  |  | Cephalosporin+Tigecycline | 4(2.2%) |
|  |  | Polymyxin+Cephalosporins+Tigacycline | 1(0.5%) |
|  |  | Polymyxin+Carbapenem+Fosfomycin | 1(0.5%) |
|  |  | Polymyxin+Carbapenem+Azactam | 1(0.5%) |
|  |  | β-lactamase inhibitors+Tigecycline | 4(2.2%) |
|  |  | Aminoglycoside+β-lactamase inhibitors | 2(1.1%) |
|  |  | Aminoglycoside+Tigecycline+Fuoroquinolones | 1(0.5%) |
|  |  | Cephalosporins+Fluoroquinolones | 1(0.5%) |
|  |  | Tigecycline+Fosfomycin | 1(0.5%) |
| A total of 78 cases of carbapenem regimen in monotherapy were treated with imipenem 43 (55.1%), biapenem 32 (41.0%) and meropenem 3 (3.8%). | | | |
| There were a total of carbapenem containing regimens in the combination treatment: 76 cases, of which 36 were imipenem (47.4%), 29 were biapenem (38.1%) and 11 were meropenem (14.5%). | | | |

| **Appendix 4. Univariate Cox regression analysis for 30-day mortality in patients with KP BSI** | | | | |
| --- | --- | --- | --- | --- |
|  | **Survived** | **Not-survived** | **HR (95% CI)** | **p-value** |
| Male sex | 63 (50%) | 32 (58%) | 1.3(0.77-2.2) | 0.32 |
| Age≥55 years | 36 (28%) | 19 (35%) | 1.2(0.67-2) | 0.6 |
| ANC＜500 neutrophils/μL | 125 (98%) | 54 (98%) | 0.82(0.11-5.9) | 0.84 |
| ANC＜100 neutrophils/μL | 95 (75%) | 43 (78%) | 1.1(0.56-2) | 0.85 |
| ANC<500/mmc for at least 15 days | 34 (27%) | 22 (40%) | 1.3(0.74-2.2) | 0.38 |
| Corticosteroid treatment | 93 (73%) | 50 (91%) | 3(1.2-7.5) | 0.02 |
| Chemotherapy | 94 (74%) | 45 (82%) | 1.2(0.6-2.4) | 0.62 |
|  |  |  |  |  |
| Acute myeloid leukemia | 84 (66%) | 34 (62%) | 0.87(0.5-1.5) | 0.61 |
| Acute lymphatic leukemia | 31 (24%) | 12 (22%) | 0.9(0.47-1.7) | 0.74 |
| lymphoma | 1 (0.8%) | 2 (3.6%) | 2.8(0.68-11) | 0.15 |
| Multiple Myeloma | 2 (1.6%) | 0 (0%) |  | 1 |
| Myelodysplastic syndrome | 9 (7.1%) | 7 (13%) | 1.6(0.71-3.5) | 0.26 |
| HSCT | 4 (3.1%) | 1 (1.8%) | 0.65(0.091-4.7) | 0.67 |
| Previous admission in ICU | 24 (19%) | 44 (80%) | 16(8-31) | ＜0.001 |
| CRKP | 28 (22%) | 31 (56%) | 3.7(2.2-6.4) | ＜0.001 |
| CVC | 17 (13%) | 11 (20%) | 1.3(0.65-2.4) | 0.5 |
| Surgery | 0 (0%) | 5 (9.1%) | 5.1(2-13) | ＜0.001 |
| CRKP | 37 (29%) | 43 (78%) | 6.7(3.5-13) | ＜0.001 |
| IIAT | 13(10.2%) | 44(80%) | 16(8.3-32) | ＜0.001 |
| Sepsis Shock | 26 (20%) | 52 (95%) | 43(13-140) | ＜0.001 |
| Combination initial antibiotic therapy | 56 (44%) | 32 (58%) | 1.6(0.94-2.7) | 0.086 |
| SOFA | 4.0 (4.0, 5.5) | 13.0 (11.0, 15.0) | 1.4(1.3-1.5) | ＜0.001 |
| CKD | 5 (3.9%) | 7 (13%) | 4.1(1.8-9.1) | ＜0.001 |
| Drinking | 6 (4.7%) | 3 (5.5%) | 1(0.33-3.4) | 0.93 |
| Smoking | 12 (9.4%) | 6 (11%) | 1(0.44-2.4) | 0.96 |
| Pitt bacteraemia score | 2.0 (1.0, 2.0) | 10.0 (6.5, 12.0) | 1.3(1.3-1.4) | ＜0.001 |
| Hypoalbuminemia | 35 (28%) | 34 (62%) | 3.7(2.1-6.3) | ＜0.001 |
| Diabetes | 4 (3.1%) | 5 (9.1%) | 2.8(1.1-7.2) | 0.026 |
| Hypotension | 29 (23%) | 52 (95%) | 40(12-130) | ＜0.001 |
| Pneumonia | 65(51.2%) | 33(60%) | 1.3(0.76-2.2) | 0.33 |
| Abbreviations: ANC: Absolute Neutrophil Count; HSCT: Hematopoietic Stem Cell Transplantation; ICU: prior Intensive Care Unit admission; CVC: Central Venous Catheter; CRKP: Carbapenem-Resistant Klebsiella pneumoniae; IIAT: Inappropriate Initial Antibiotic Therapy; CKD: Chronic Kidney Disease. | | | | |
